# Cerebrospinal fluid analysis in 108 patients with progressive multifocal leukoencephalopathy

**DOI:** 10.1186/s12987-020-00227-y

**Published:** 2020-10-27

**Authors:** Nora Möhn, Yi Luo, Thomas Skripuletz, Philipp Schwenkenbecher, Anne Ladwig, Clemens Warnke, Sven G. Meuth, Heinz Wiendl, Catharina C. Gross, Christoph Schröder, Aiden Haghikia, Martin Stangel

**Affiliations:** 1 Clinical Neuroimmunology and Neurochemistry, Department of Neurology, Hannover Medical School, Carl-Neuberg-Str. 1, 30625 Hannover, Germany; 2 Department of Neurology, University of Cologne, Faculty of Medicine and University Hospital Cologne, Cologne, Germany; 3 Department of Neurology with Institute of Translational Neurology, University Hospital Münster, Münster, Germany; 4 Department of Neurology, Ruhr-University Bochum, St. Josef-Hospital Bochum, Bochum, Germany

**Keywords:** Progressive multifocal leukoencephalopathy, Cerebrospinal fluid, Multiple sclerosis, HIV, Lymphoma

## Abstract

**Background:**

Progressive multifocal leukoencephalopathy (PML) is caused by an opportunistic infection with JC polyoma virus (JCPyV) and mainly affects immunocompromised patients. It leads to pronounced demyelination of the central nervous system (CNS) resulting in severe disability or even death. Detection of JCPyV DNA in the cerebrospinal fluid (CSF) is usually accepted as proof for the diagnosis of PML. Routine CSF parameters, like CSF cell count, protein concentration, Qalbumin, or intrathecal immunoglobulin synthesis are mostly considered normal. However, this has not been investigated systematically.

**Methods:**

We analyzed routine CSF parameters in a cohort of 108 PML patients that were treated at four different neurological centers in Germany. The patients exhibited different underlying conditions with natalizumab-treated multiple sclerosis (n = 54) and human immunodeficiency virus (HIV)-infection (n = 25) being the most frequent. The data were collected at the respective centers in accordance with local requirements and then jointly analyzed. The total PML cohort was compared with a control group of patients with normal pressure hydrocephalus (NPH) and idiopathic intracranial hypertension (IIH). Multiple sclerosis and HIV patients were additionally compared with their own non-PML control groups.

**Results:**

The PML group showed an elevated cell count (p < 0.001) compared to the control group, however, this effect was mainly driven by HIV-PML patients. This subgroup also demonstrated a significantly higher proportion of patients with a disturbed blood-CSF-barrier function.

**Conclusions:**

This comprehensive, retrospective study on CSF diagnostic analysis in PML patients provides insight into the CSF of those patients. It demonstrates that CSF composition in PML patients may be specific for the underlying condition that predisposes for the development of PML and thus data have to be interpreted in this context.

## Background

Progressive multifocal leukoencephalopathy (PML) is a demyelinating disease of the central nervous system (CNS) caused by reactivation of JC polyoma virus (JCPyV) and finally leading to a predominant destruction of oligodendrocytes [1]. JCPyV is an opportunistic pathogen; thus, it primarily occurs in immune-compromised patients. The first pathological description of this disease was in 1958, whereby the affected patient suffered from chronic lymphatic leukemia and Hodgkin’s disease [2]. The first human immunodeficiency virus (HIV)-infected patient with PML was reported in 1982 [3]. PML is also a well-known serious adverse event in natalizumab-treated multiple sclerosis (MS) patients [4, 5]. Other conditions predisposing to PML are hematologic malignancies, post-transplant immunosuppression, or other diseases requiring immunosuppressive/immunomodulatory drugs (such as rituximab, cyclophosphamide, methotrexate, dimethyl fumarate, ciclosporin, mycophenolate mofetil, or fingolimod) [6–8]. Only in few instances, can no apparent immunosuppression be found [9], albeit immune senescence may play a role in such cases. PML is a rare disease, but it is characterized by high mortality rates and long-term neurologic morbidity. In order to ensure a validated PML diagnosis, clinical, imaging, and laboratory features are needed [10]. The detection of JCPyV DNA in CSF in combination with appropriate clinical symptoms and radiological characteristics allow a definite diagnosis of PML without biopsy. In individual cases, i.e. if JCPyV DNA remains undetectable in the CSF, the presence of characteristic pathoanatomic findings from a CNS biopsy specimen may be required to establish diagnosis [10]. No evidence-based therapeutic options are available and reconstitution of the immune system, e.g. by withdrawal of immunosuppressive drugs or provision of antiretroviral therapy, are the only therapies that have demonstrated survival benefit [11]. More recently, favorable outcomes have been reported in PML patients treated with immune checkpoint inhibitor therapies [12–14] and infusion of virus-specific T cells [15]. Without doubt, an early diagnosis and consequently a prompt start of therapy/cessation of causal immune suppression could improve the prognosis. Unfortunately, the diagnosis of PML is often delayed. Potential reasons are the lack of specific radiographic features in brain magnetic resonance imaging (MRI) and the fact that CSF JCPyV-PCR may be negative, particularly at an early stage of the disease. The search for a potential blood- or CSF-biomarker for PML, especially in CSF negative patients, has not been successful so far [16]. Generally, there is a fundamental lack of information on routine CSF parameters in PML patients. Even though large cohorts of PML patients under natalizumab treatment have been described [17, 18] and a large retrospective, observational study about risk factors and outcomes of PML patients has been published recently [19] results about basic CSF parameters have not been published in detail. Especially in times of promising, new therapeutic options, knowledge about distinct diagnostic features of CSF analysis is important to define risk groups or to evaluate treatment response. Here, we report the results of routine CSF parameters from 108 PML patients that were treated at four University hospitals in Germany. Data obtained was further linked to patient clinical data and their JCPyV PCR results.

## Methods

### Study design and setting

Patients were recruited from the Department of Neurology at Hannover Medical School (n = 50), St. Josef Hospital Bochum (n = 38), University Hospital Münster (n = 19), and University Hospital Cologne (n = 12). Five patients with HIV infection were excluded for further analysis because three were diagnosed with additional cerebral toxoplasmosis and in two patients PML diagnosis was only suspected. Another six patients had to be excluded due to insufficient data quality, resulting in n = 108 patients available for final analysis (Additional file 1). Patients with PML, diagnosed with other viral CNS infections such as varicella zoster virus (VZV)-, herpes simplex virus (HSV)-, Epstein-Barr virus (EBV)-, or cytomegalovirus (CMV) encephalitis were excluded. The CSF of the PML cohort was compared with the CSF of an age-matched control group consisting of patients with normal pressure hydrocephalus (NPH) (n = 8) and idiopathic intracranial hypertension (IIH) (n = 13) (Additional file 2). In addition, the data of the HIV subgroup were compared with a non-PML HIV control group (n = 37) where patients had a lumbar puncture for other reasons, for example cognitive deficits, suspected encephalitis, suspected vasculitis, unexplained encephalopathy, or seizures (Additional file 3). Another control group consisted of aged-matched multiple sclerosis patients without PML (n = 54) who received CSF analysis as part of the diagnostic process (Additional file 4).

### Diagnostic procedures

CSF and serum were analyzed by routine methods [20]. CSF cell count, total protein, and lactate were analyzed immediately after CSF withdrawal by lumbar puncture. CSF cells were counted manually with a Fuchs-Rosenthal counting chamber. For further analyses the residual CSF was centrifuged (145 g for 15 min) and the supernatant frozen at − 70 °C. The function of the blood-CSF barrier was estimated as CSF/serum albumin quotients (Qalbumin). The age-adjusted upper reference limit of Qalbumin (Qalb) was calculated using the formula Qalb = 4 + (age in years/15) [21]. CSF oligoclonal bands were determined by isoelectric focusing in polyacrylamide gels with consecutive silver staining. Five patterns of oligoclonal bands were distinguished following the recommendations of the first European consensus on CSF analysis in multiple sclerosis [22]. CSF acquisition, CSF processing and storage as well as laboratory methods were similar across the different institutions and were carried out according to the guidelines of the German society for CSF diagnostics and clinical neurochemistry [23]. Furthermore, the acquisition and processing of lumbar CSF in NPH and IIH patients who served as a control group was comparable to acquisition and processing in PML patients.

### Statistical analysis

Statistical analysis was performed using GraphPad Prism software version 8.0 (GraphPad Software, San Diego, USA). The Mann–Whitney U-test as the nonparametric equivalent of the t-test for independent samples was used to compare data between two groups. For this test the data does not need to be normally distributed, the variables only need to be ordinally scaled. A one-way analysis of variance (ANOVA) with a Tukey’s multiple comparisons test was used to analyze data of multiple groups. Data are displayed as means and standard error of the mean (SEM) or standard deviation (SD), respectively. P < 0.05 was considered statistically significant.

## Results

### Patient characteristics and routine CSF parameters

In total, the CSF results of 108 patients from four centers were analyzed. The patients’ mean age was 48 years, with a range from 19 to 81 years. The underlying diagnoses included multiple sclerosis treated with natalizumab (MS/NTZ, n = 54), HIV infection (n = 25), and hematological diseases (n = 19) such as B-cell non-Hodgkin lymphoma (n = 10), multiple myeloma (n = 2), chronic lymphocytic leukemia (n = 6), and acute myeloid leukemia (n = 1) (referred to collectively as lymphoma group). Ten patients of the lymphoma group were treated with rituximab mono- or combination-therapy. Other chemotherapy regimens included bendamustine, melphalane, mitoxantrone, or methotrexate. In four of the patients an organ transplant had been performed and they received immunosuppressive therapy with tacrolimus, mycophenolate mofetil, or ciclosporin. One patient each suffered from bronchial carcinoma, sarcoidosis, microscopic polyangiitis and common variable immunodeficiency (CVID). In two cases no explanatory underlying disease was found. Detailed information about the individual patients can be found in Additional file 4. Compared to the routine CSF parameters (Table 1), 24/108 patients (22%) had an elevated cell count and 35/108 patients showed an elevated Qalbumin indicating a disturbed blood-CSF-barrier. The mean lactate content was 1.64 mmol/l (range: 0.99–2.8 mmol/l), whereby 6/108 (6%) subjects presented with increased lactate levels.

**Table 1:** Routine CSF parameters of diagnostic LP (whole cohort)

	Standard value [24]	Elevated (n)	Mean	Maximum	Minimum
Cell count (cell/µl)	≤ 4	24	5	34	1
Qalbumin	< 7.3^a^	35	8.62	25.1	1.9
CSF protein (mg/l)	≤ 500	41	541	1330	226
CSF lactate (mmol/l)	< 2.2	6	1.64	2.8	0.99

*CSF* cerebrospinal fluid

^a^With an average age of the cohort of 48 years, the upper limit of Qalbumin is 7.2 (Qalbumin = 4 + (age/15)

### Comparison of PML cohort and control group

**Fig. 1:**
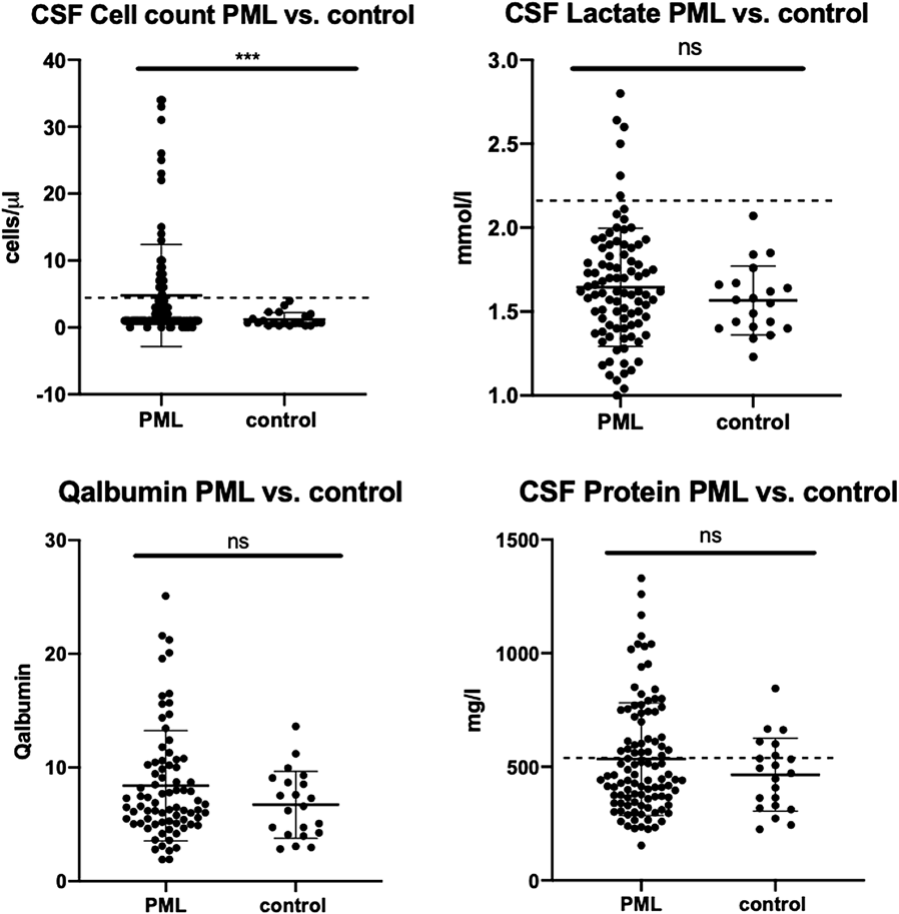
Comparison of CSF cell count, lactate, Qalbumin, and CSF protein of all PML patients with control (NHP and IIH) patients. PML: progressive multifocal leukoencephalopathy. Data is presented as mean ± standard deviation. Levels of significance: ***p < 0.001, two-tailed p-value, Mann–Whitney-test with comparison of median values was applied. ns: not significant

Routine CSF parameters of the whole PML cohort were compared with CSF results of an aged matched control group (Additional file 2: Table S2) consisting of patients with NPH (n = 8) or IIH (n = 13). In the case of CSF lactate, Qalbumin, and CSF protein there was no difference between the two groups. In contrast, the CSF mean cell count of the PML cohort was significantly higher compared with the control group (p < 0.001, Fig. 1).

### Comparison of different PML subgroups regarding routine CSF parameters at diagnostic lumbar puncture

**Fig. 2:**
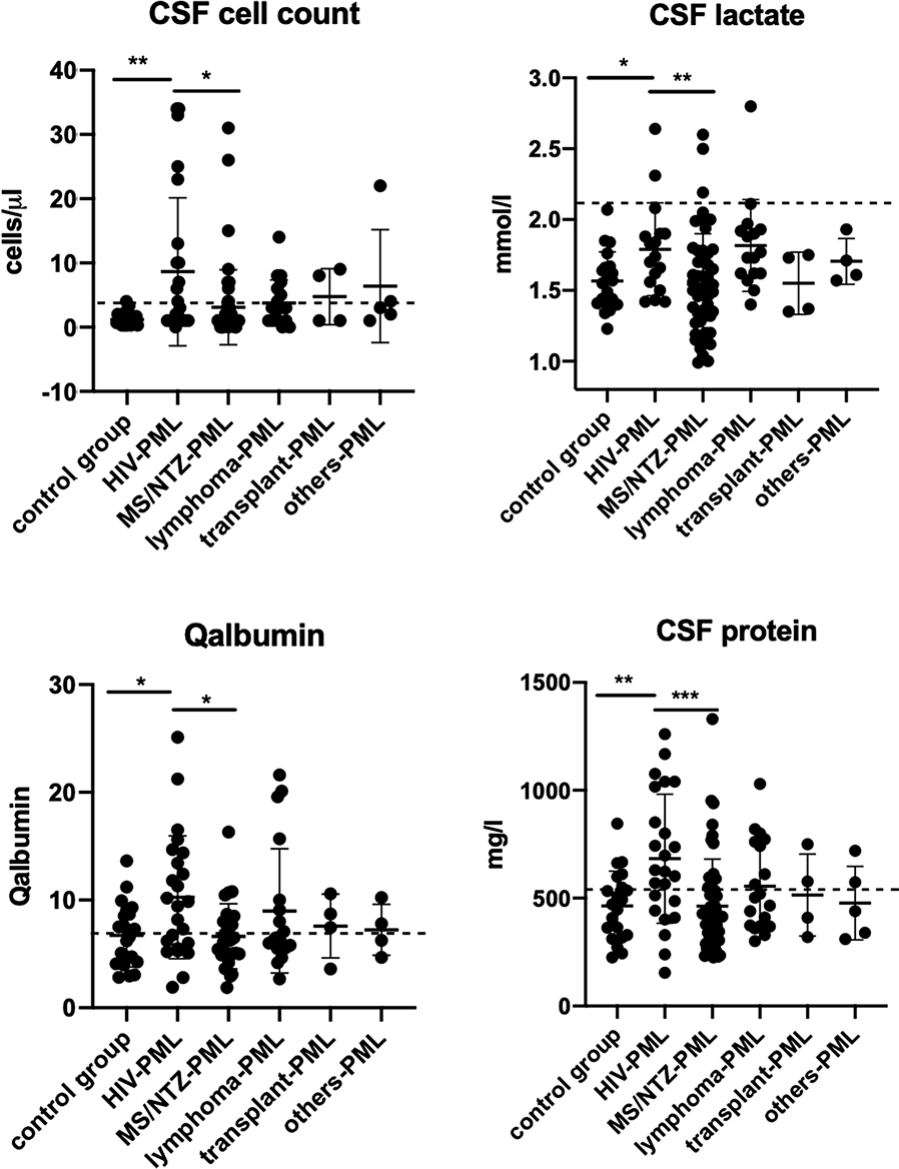
Comparison between different PML subgroups and control group (NPH and IIH) regarding CSF cell count, Qalbumin, CSF protein, and CSF lactate. CSF: cerebrospinal fluid, HIV: human immunodeficiency virus, MS: multiple sclerosis. Data is presented as mean ± standard deviation. Levels of significance: ***p < 0.001, **p < 0.01, *p < 0.05, two-tailed p-value, Mann–Whitney-test with comparison of median values was applied to compare data of two groups. One-way analysis of variance (ANOVA) with a Tukey’s multiple comparisons test were used to analyze data of multiple groups

We then compared the PML patients with different underlying diagnoses (Fig. 2). Patients with PML together with HIV infection showed a higher cell count, CSF lactate, CSF protein, and Qalbumin level in comparison with the control group. Other PML subgroups compared among each other and compared with the control group were not significantly different from the routine CSF parameters. The HIV PML patients exhibited a higher cell count, CSF protein, Qalbumin, and CSF lactate compared to MS patients who had PML because of natalizumab treatment.

### Comparison between HIV control group and HIV-PML group

**Fig. 3:**
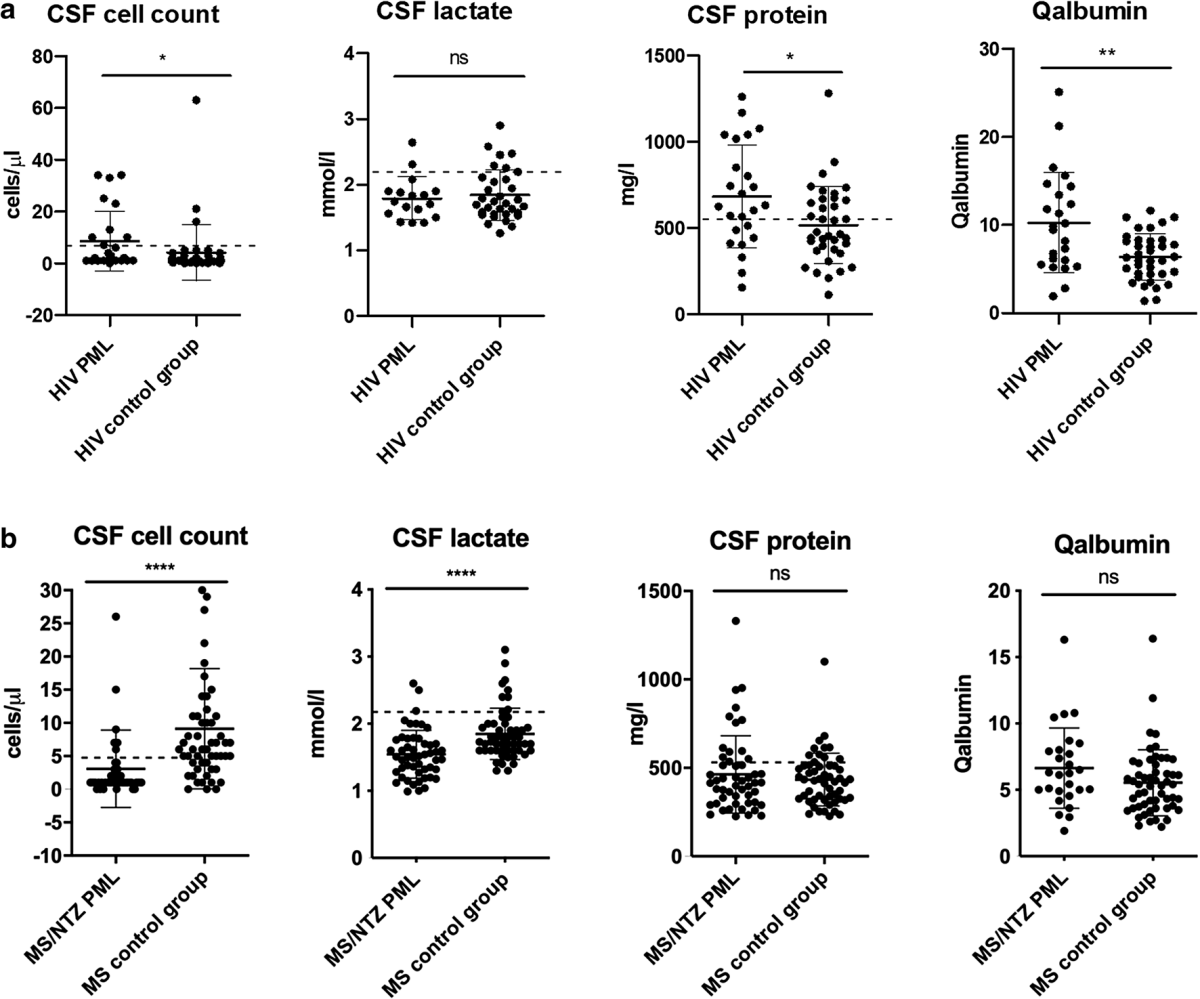
Comparison of PML patients and respective control groups. **a** HIV PML patients compared with HIV patients without PML regrading CSF cell count, CSF lactate, CSF protein and Qalbumin. **b** MS/NTZ patients compared with MS control group. CSF: cerebrospinal fluid, HIV: human immunodeficiency virus, MS: multiple sclerosis, NTZ: natalizumab, PML: progressive multifocal leukoencephalopathy. Data is presented as mean ± standard deviation. Levels of significance: ****p < 0.0001, **p < 0.01, *p < 0.05, ns: not significant, two-tailed p-value, Mann–Whitney-test with comparison of median values was applied

To investigate whether the differences between HIV PML patients and the other subgroups regarding routine CSF parameters were caused by either the HIV infection itself or by the PML, the HIV PML patients were compared with an HIV control group without PML (Additional file 3: Table S3). While there was no significant difference concerning CSF lactate, HIV-PML patients showed a significantly higher CSF cell count (P < 0.05, CSF protein (P < 0.05) and Qalbumin (P < 0.01) compared with the non-PML control group (Fig. 3a). MS/NTZ PML patients were also compared with a respective control group (Fig. 3b). MS control patients exhibited a significantly higher CSF cell count (P < 0.001). This is most likely explained by the fact that control group patients were mostly untreated, as the lumbar puncture was performed during the diagnostic process for MS. CSF lactate concentration was also significantly higher in the control group compared with the MS/NTZ PML group (P < 0.001) while there was no difference between MS/NTZ PML and control group patients regarding CSF protein or albumin quotient.

### CSF cell distribution in PML patients

In 80 patients of the total cohort a differentiation of cell distribution was performed during CSF analysis. Sixty-seven patients (79%) showed a lymphocytic predominance while in 10 patients (12%) the majority of cells was monocytic. Six patients (7%) exhibited a mixed cell distribution and only two patients (2%) demonstrated mainly granulocytes within the CSF. The latter is best explained by blood contamination. Considering the individual subgroups, patients of the MS/NTZ-, the HIV-, the lymphoma-, and the transplant-group all showed a lymphocytic predominance (Table 2) as did all the control groups. Some of the HIV patients showed a mixed cell distribution, however, all of these had blood contaminations in the lumbar puncture.

**Table 2:** CSF cell distribution of PML subgroups

MS/NTZ group (n = 48)	HIV group (n = 15)	Lymphoma group (n = 10)	Transplant group (n = 7)	NPH + IIH group (n = 21)	HIV control group (n = 37)	Ms control group (n = 54)
Lymphocytic predominance in 84% Granulocytic predominance in 4% Monocytic predominance in 4% Mixed cell distribution in 8%	Lymphocytic predominance in 87% Monocytic predominance in 6.5% Mixed cell distribution in 6.5%	Lymphocytic predominance in 65% Monocytic predominance in 29% Mixed cell distribution in 6%	Lymphocytic predominance in 86% Monocytic predominance in 14%	Lymphocytic predominance in 95% Monocytic predominance in 5%	Lymphocytic predominance in 85% Monocytic predominance in 3% Mixed cell distribution in 12%	Lymphocytic predominance in 98% Monocytic predominance in 2%

*HIV* human immunodeficiency virus, *IIH* idiopathic intracranial hypertension, *MS* multiple sclerosis. *NPH* normal pressure hydrocephalus, *NTZ* natalizumab. Normal cell distribution is defined as 90–60% lymphocytes and 10–40% monocytes

### Analysis of oligoclonal bands of PML patients

At diagnostic lumbar puncture, oligoclonal bands (OCB) were analyzed in 58 patients. In 22 cases (38%) OCB type 2 (OCB in CSF only) were found, of which 18 patients belonged to the MS/NTZ group and one patient each to the HIV-, lymphoma-, and transplant-group. Nine patients (16%) exhibited OCB type 3 (identical OCB in CSF and serum and additional OCB in CSF only). The majority of patients (n = 5) suffered from HIV as underlying disease, two patients had MS/NTZ and one patient each belonged to the lymphoma and transplant group. Oligoclonal bands were negative in 27 patients, with 13 patients (22%) showing type 1 OCB (no OCB) and 14 patients (24%) showing type 4 OCB (identical OCB in CSF and serum). Seven patients (26%) of the MS/NTZ group exhibited negative oligoclonal bands. This effect might be due to the natalizumab treatment which is known to modify oligoclonal bands [25, 26].

## Discussion

Here, we present our results regarding the analysis of routine CSF parameters in a cohort of 108 PML patients. Compared with a control group consisting of patients diagnosed with NPH or IIH, our cohort exhibited a significantly higher CSF cell count. However, this was mainly due to the HIV-PML group, while the CSF cell counts were normal in the other PML subgroups (Fig. 2). CSF protein and Qalbumin as an indicator for the integrity of the blood-CSF barrier function were not significantly different between PML and control groups (Fig. 1). Compared with the control group and the MS/NTZ-PML subgroup the HIV-PML patients had a significantly higher CSF protein, Qalbumin, and CSF lactate (Fig. 2). It is likely that these inflammatory changes in HIV patients are due to the HIV infection itself. However, cell count of HIV-patients with validated PML was also elevated in comparison with patients of the HIV control group (Fig. 3). Previous studies have shown that HIV infection is frequently accompanied by CSF pleocytosis occurring early in the infection that often resolves with antiretroviral therapy [27–29]. A detailed analysis of the cellular composition of CSF in HIV-infected subjects via flow cytometry demonstrated that these patients exhibit an increase in the absolute number of CSF T cells and an even higher increase in the absolute number of CSF CD8+ T cells compared with a non-infected control-group [30]. The inflammatory processes in PML seem to lead to a further increase in CSF cell count in HIV patients and one can only speculate about the reasons for this. In addition to the cell count, protein and Qalbumin levels of the HIV-PML group were significantly elevated compared to the respective control group as well (Fig. 3). This indicates that the presence of PML in HIV patients leads to an increased dysfunction of the blood-CSF barrier. In summary, the majority of PML patients show inconspicuous results on CSF testing. In the subgroup of HIV patients, however, increased cell counts as well as elevated CSF protein and Qalbumin levels are regularly observed. Pleocytosis may be an effect of the CNS involvement of the HIV infection itself, but just like CSF protein concentrations and Qalbumin levels, CSF cell count in HIV patients might also increase due to PML.

The detection of OCB at the initial lumbar puncture was highly dependent on the underlying disease. Thus, the majority of patients with intrathecal immunoglobulin synthesis belonged to the MS/NTZ-subgroup. However, some patients of the other groups, especially HIV infected patients, exhibited positive OCB as well. This is consistent with the observation that HIV infection induces a humoral immune response in the CNS, as measured by an increased intrathecal IgG production, in both neurologically asymptomatic and symptomatic patients [29, 31].

Our study is limited by its retrospective approach and the heterogenous clinical data from different institutions. The cohort includes patients with different etiology of diseases and various administrated therapies. Individual subgroups were partly small and especially patients of the HIV subgroup exhibited a certain heterogeneity regarding duration of HIV infection or antiretroviral therapy. Due to the nature of this investigation, interpreting causal relations is difficult. However, to our knowledge this is the largest study on routine CSF parameters in PML patients with different underlying conditions.

## Conclusion

CSF analysis is routinely performed for the differential diagnosis of PML. The detection of JCPyV DNA in the CSF is the decisive step in the diagnosis of this disease. So far it was assumed that CSF routine parameter are normal in PML, however, our data show that an elevated cell count does not rule out PML, especially in patients with underlying HIV infection. These patients may also exhibit a disturbed blood-CSF barrier. Additionally, immunoglobulin production and oligoclonal bands also depend on the underlying disease entity i.e. like a type 2/3 pattern in MS. Thus, CSF data in patients with suspected PML have to be carefully interpreted in the context of the underlying cause of the immunodeficiency, comorbidities and concomitant treatments.

## Supplementary information

**Additional file 1.** Patients’ characteristics and CSF parameter of PML patients. Description: Table is divided into individual subgroups (multiple sclerosis, HIV, lymphoma, transplant, others). AML: acute myeloid leukemia; BEACOPP: bleomycin + etoposide + adriamycin + cyclophosphamide + oncovin + procarbazine + prednisolone; CHOP: cyclophosphamide + doxorubicin + oncovin + prednisolone; CLL: chronic lymphocytic leukemia; CSF: cerebrospinal fluid; CVID: common variable immunodeficiency; DHAP: rituximab + dexamethasone + high dose Ara-C + cisplatin; DLBCL: diffuse large b-cell lymphoma; HIV: human immunodeficiency virus; MMF: mycophenolate mofetil; MTX: methotrexate; n.a.: not applicable; NHL: non-Hodgkin lymphoma; OCB: oligoclonal bands; PML: progressive multifocal leukoencephalopathy; RRMS: relapsing remitting multiple sclerosis; T-LGL-leukemia: T-large granular lymphocytes-leukemia.

**Additional file 2.** Patients’ characteristics and routine CSF parameter of control group patients. Description: CSF: cerebrospinal fluid; f: female; IIH: idiopathic intracranial hypertension; LP: lumbar puncture; m: male; NPH: normal pressure hydrocephalus; OCB: oligoclonal bands.

**Additional file 3.** Patients’ characteristics and routine CSF parameter of HIV control group patients. Description: CSF: cerebrospinal fluid; f: female; LP: lumbar puncture; m: male; n.a.: not applicable; OCB: oligoclonal bands. The suspected diagnoses as reasons for lumbar punctures included cognitive retardation, suspected encephalitis, suspected vasculitis, unexplained encephalopathy, and seizures.

**Additional file 4.** Patients’ characteristics and routine CSF parameter of MS control group patients. Description: CSF: cerebrospinal fluid; f: female; LP: lumbar puncture; m: male; n.a.: not applicable; OCB: oligoclonal bands.

## Data Availability

The datasets used and/or analysed during the current study are available from the corresponding author on reasonable request.

## Abbreviations

CMV: Cytomegalovirus
CNS: Central nervous system
CSF: Cerebrospinal fluid
EBV: Epstein-Barr virus
HIV: Human immunodeficiency virus
HSV: Herpes simplex virus
IIH: Idiopathic intracranial hypertension
IRIS: Immune reconstitution syndrome
JCPyV: JC polyoma virus
MRI: Magnetic resonance imaging
MS: Multiple sclerosis
NPH: Normal pressure hydrocephalus
NTZ: Natalizumab
PML: Progressive multifocal leukoencephalopathy
VZV: Varicella zoster virus
