# Supplementary material for: Cerebrospinal fluid analysis in 108 patients with progressive multifocal leukoencephalopathy
Source: Fluids Barriers CNS. 2020 Oct 27;17:65. doi: 10.1186/s12987-020-00227-y (PMC7590675; doi:10.1186/s12987-020-00227-y)
Supplement: Supplementary file 1 — Additional file 1. Patients’ characteristics and CSF parameter of PML patients. Description: Table is divided into individual subgroups (multiple sclerosis, HIV, lymphoma, transplant, others). AML: acute myeloid leukemia; BEACOPP: bleomycin + etoposide + adriamycin + cyclophosphamide + oncovin + procarbazine + prednisolone; CHOP: cyclophosphamide + doxorubicin + oncovin + prednisolone; CLL: chronic lymphocytic leukemia; CSF: cerebrospinal fluid; CVID: common variable immunodeficiency; DHAP: rituximab + dexamethasone + high dose Ara-C + cisplatin; DLBCL: diffuse large b-cell lymphoma; HIV: human immunodeficiency virus; MMF: mycophenolate mofetil; MTX: methotrexate; n.a.: not applicable; NHL: non-Hodgkin lymphoma; OCB: oligoclonal bands; PML: progressive multifocal leukoencephalopathy; RRMS: relapsing remitting multiple sclerosis; T-LGL-leukemia: T-large granular lymphocytes-leukemia. [file 12987_2020_227_MOESM1_ESM.pdf]

Multiple sclerosis patients

Pat.	Age at diagnosis PML	underlying disease	immunosuppressive therapy	cell count	cytology	lymphocytes (%)	monocytes (%)	others (%)	OCB	Qalbumin	CSF protein (mg/l)	CSF lactate (mmol/l)	CSF JCV PCR (c/ml)	additional findings
**1**	43	RRMS	natalizumab	1	inconspicuous	60	40		type 4	10.79	841	1.38	1080	
**2**	42	RRMS	natalizumab, since 2010	2	inconspicuous	80	20		type 2	2.94	296	1.49	400	
**3**	32	RRMS	natalizumab, since 07/2011	1	inconspicuous	80	20		type 2	5.04	331	1.56	700	
**4**	55	RRMS	natalizumab	1	inconspicuous	80	20		type 2	6.12	396	1.94	800	
**5**	36	RRMS	natalizumab	2	activated	50	41	9	type 2	3.63	305	1.52	n.a.	
**6**	32	RRMS	natalizumab	1	granulocytic	0		100	n.d.	n.d.	229	1.27	n.a.	
**7**	40	RRMS	natalizumab	1	lymphocytic	100			n.d.	n.d.	300	1.79	n.a.	
**8**	33	RRMS	natalizumab	9	mainly lymphocytic	76			type 2	n.d.	940	1.32	n.a.	
**9**	46	RRMS	natalizumab	4	mainly granulocytic	33		67	n.d.	n.d.	561	2.05	n.a.	
**10**	38	RRMS	natalizumab	1	lymphocytic	100			n.d.	n.d.	291	1.32	n.a.	
**11**	30	RRMS	natalizumab	1	lymphocytic/granulocytic	50		50	n.d.	n.d.	791	1.35	n.a.	
**12**	45	RRMS	natalizumab	3	mainly lymphocytic	87,5			type 2	n.d.	536	1.99	n.a.	
**13**	46	RRMS	natalizumab	2	mainly lymphocytic	66			n.d.	n.d.	378	1.78	n.d.	
**14**	34	RRMS	natalizumab	1	lymphocytic	100			n.d.	n.d.	259	1.00	3000	
**15**	40	RRMS	natalizumab	7	mainly lymphocytic	90			n.d.	n.d.	515	1.28	n.a.	
**16**	43	RRMS	natalizumab	1	lymphocytic	100			n.d.	n.d.	952	1.19	n.a.	
**17**	41	RRMS	natalizumab	1	lymphocytic	100			type 2	n.d.	417	1.60	negative	
**18**	35	RRMS	natalizumab	1	lymphocytic	100			n.d.	n.d.	345	1.12	positive	
**19**	38	RRMS	natalizumab	2	lymphocytic	100			type 2	n.d.	446	1.09	negative	
**20**	52	RRMS	natalizumab	1	lymphocytic	100			n.d.	n.d.	340	1.78	193.830	
**21**	27	RRMS	natalizumab	1	lymphocytic	100			type 1	n.d.	259	0.99	positive	
**22**	42	RRMS	natalizumab	3	lymphocytic	75			n.d.	n.d.	289	1.20	2170	
**23**	19	RRMS	natalizumab	6	lymphocytic	100			n.d.	n.d.	366	1.13	positive	

**24**	34	RRMS	natalizumab	2	lymphocytic	100			type 2	n.d.	364	1.68	positive
**25**	46	RRMS	natalizumab	15	n.d.	n.d.			Typ 1	n.d.	460	2.5	positive
**26**	60	RRMS	natalizumab	3	mainly lymphocytic	80			n.d.	n.d.	465	1.04	
**27**	39	RRMS	natalizumab	1	lymphocytic	100			n.d.	n.d.	463	1.51	
**28**	51	RRMS	natalizumab	1	lymphocytic	100			n.d.	n.d.	226	1.47	
**29**	36	RRMS	natalizumab	1	lymphocytic	100			type 2	4.18	266	1.46	
**30**	46	RRMS	natalizumab	1	lymphocytic	100			n.d.	n.d.	235	1.34	
**31**	34	RRMS	natalizumab	1	lymphocytic	100			type 1	4.54	298	1.46	
**32**	42	RRMS	natalizumab	2	mainly lymphocytic	57			n.d.	5.43	399	1.36	
**33**	62	RRMS	natalizumab	1	lymphocytic	100			type 3	10.46	613	1.61	
**34**	54	RRMS	natalizumab	n.d.	n.d.	n.d			n.d.	n.d.	n.d.	n.d.	
**35**	52	RRMS	natalizumab	1	lymphocytic	100			type 2	7.38	414	1.18	
**36**	40	RRMS	natalizumab	2	lymphocytic	100			type 1	7.9	590	1.6	
**37**	48	RRMS	natalizumab	1	lymphocytic	100			n.d.	6.24	514	1.2	
**38**	33	RRMS	natalizumab	7	mainly lymphocytic	95			type 2	5	427	1.58	
**39**	47	RRMS	natalizumab	n.d.	n.d.	n.d			n.d.	n.d.	n.d.	n.d.	
**40**	33	RRMS	natalizumab	1	lymphocytic	100			n.d.	6.5	375	1.15	
**41**	49	RRMS	natalizumab	4	lymphocytic	100			type 3	8.5	596	1.4	
**42**	46	RRMS		31	lymphocytic	100			n.d.	10.7	770	1.6	
**43**	48	RRMS	azathioprine; natalizumab (5 y, 9 m)	26	lymphocytic	94	6		type 2	5	443	2	
**44**	37	RRMS	natalizumab (5 y)	0	lymphocytic	71	7		type 2	4.9	428	1.54	
**45**	33	RRMS	natalizumab (4.5 y)	1	lymphomonocytic	49	27		n.d.	3.1	265	1.65	
**46**	45	RRMS	natalizumab (100 infusions)	0	lymphomonocytic	49	23	9	type 4	16.3	1330	1.76	
**47**	33	RRMS	natalizumab (26 infusions)	0	lymphocytic	65	8	13	type 1	1.9	233	2	
**48**	25	RRMS	natalizumab (4 y, 50 infusions)	0	lymphocytic	69	22	4	type 2	8.7	755	2.19	
**49**	52	RRMS	natalizumab	0		0	0		type 2	6.3	440	1.5	

**50**	48	RRMS	natalizumab (36 infusions)	1		0	100			6.9	510	1.7		
**51**	44	RRMS	natalizumab (2007-2012)											
**52**	50	RRMS	natalizumab (26 infusions)	1		100	0		type 2	5.1	380	1.7		
**53**	41	RRMS	natalizumab (2009-2015)	0		0	0			7.7	550	1.8		
**54**	50	RRMS	Avonex, natalizumab	1		0	100		type 2	8	415	2.6		

HIV patients

**Pat.**	**Age at diagnosis PML**	**underlying disease**	**immunosuppressive therapy**	**cell count**	**cytology**	**lymphocytes (%)**	**monoctes (%)**	**others (%)**	**OCB**	**Qalbumin**	**CSF protein (mg/l)**	**CSF lactate (mmol/l)**	**CSF JCV PCR (c/ml)**	**additional findings**
**1**	31	HIV, Hodgkin lymphoma	chemotherapy (BEACOPP)	33	activated	100	0		type 3	13.45	1168	2.31	1080	
**2**	47	HIV	none	2	inconspicuous	85	10	5	n.a.	6.76	442	1.62	400	
**3**	50	HIV	none	1	inconspicuous	90	10		type 3	9.45	487	1.66	700	
**4**	57	HIV	none	25	activated	80	17	3	type 3a	21.24	1017	2.08	800	
**5**	43	NHL, HIV	n.a.	10	slightly activated	75	25		type 2	6.2	513	n.d.	n.a.	
**6**	37	HIV	none	34	activated	n.a.	n.a.		type 4	15.6	1040	1.84	n.a.	
**7**	n.a.	HIV	none	n.a.	n.a.	n.a.	n.a.		n.a.	n.a.	n.a.	n.a.	n.a.	
**8**	n.a.	HIV	none	n.a.	n.a.	n.a.	n.a.		n.a.	n.a.	n.a.	n.a.	n.a.	
**9**	39	HIV	none	1	inconspicuous	n.a.	n.a.		type 2	6	402	1.83	n.a.	
**10**	36	HIV	none	1	inconspicuous	n.a.	n.a.		n.a.	14.69	800	n.a.	n.a.	
**11**	73	HIV	none	1	n.a.	n.a.	n.a.		type 3	25.1	1076	2.64	n.a.	
**12**	40	HIV	none	3	slightly activated	n.a.	n.a.			5.29	410	1.7	n.a.	
**13**	28	HIV	none	n.d.	n.d.	n.d.	n.d.	n.d.	n.d.	n.d.	n.d.	n.d.	n.d.	histological diagnostic confirmation
**14**	62	HIV	none	6	activated	90	10		type 4	12.4	851	n.a.	3000	
**15**	53	HIV	none	1	n.a.	n.a.	n.a.			2.81	239	n.a.	n.a.	
**16**	40	HIV	none	2	n.d.	n.d.	n.d.		type 2	11.8	602	n.a.	n.a.	
**17**	49	HIV	none	13	n.a.	n.a.	n.a.		n.a.	n.a.	n.a.	n.a.	negative	histological diagnostic confirmation

**18**	46	HIV	none	2.3	n.a.	n.a.	n.a.		type 4	5.2	566	1.88	positive	
**19**	47	HIV	none	7	activated	95	5		type 3	14.38	737	1.43	negative	
**20**	51	HIV		1	n.d.	n.d.	n.d.		type 1	7.3	623	1.74	193.830	
**21**	42	HIV		1	lymphocytic	78	2	1	n.d.	16.5	1260	1.42	positive	
**22**	40	HIV		4	lymphocytic	51	8	9	type 1	8.2	630	1.9	2170	
**23**	41	HIV		0	lymphocytic	79	2	10	type 2	5.5	698	1.42	positive	
**24**	48	HIV	-	34			100		type 3	11.3	1040	1.9	positive	
**25**	54	HIV	-	1		0	100			10.2	570	1.5	positive	

Patients with hematological malignancies (lymphoma group)

**Pat.**	**Age at diagnosis PML**	**underlying disease**	**immunosuppressive therapy**	**cell count**	**cytology**	**lymphocytes (%)**	**monocytes (%)**	**others (%)**	**OCB**	**Qalbumin**	**CSF protein (mg/l)**	**CSF lactate (mmol/l)**	**CSF JCV PCR (c/ml)**	**additional findings**
**1**	77	NHL	rituximab	3	inconspicuous	75	25		type 4	6.03	369	n.a.	200.000	
**2**	69	NHL	rituximab, bendamustine	n.a.	n.a.	n.a.	n.a.		n.a.	n.a.	n.a.	n.a.	2890	
**3**	55	CLL	rituximab	7	activated	89	11		type 4	10.02	742	1.93	500	
**4**	73	NHL	rituximab	1	inconspicuous	80	20		type 2	2.69	301	1.4	negative	
**5**	77	B-CLL	rituximab	8	inconspicuous	90	10		type 1	4.65	374	1.88	250	
**6**	61	NHL	n.a.	4	slightly activated	90	10		type 4	9.1	563	1.62	n.a.	
**7**	54	multiple myeloma	multiple chemotherapy (cyclophosphamide, bendamustine, and others), 5x autologous PBSCT	1	inconspicuous	n.a.	n.a.		type 4	19.59	820	1.73	<180	
**8**	63	T-LGL-leukemia	MTX	1	slightly activated	80	20		type 3	6.49	440	1.92	203	
**9**	69	NHL	rituximab, bendamustine	3	slightly activated	78	22		type 2a	4.18	329		90.000	
**10**	73	NHL	mitoxantrone, melphalan, prednisolone	8	slightly activated	44	56		type 3	21.6	773	1.73	negative	histological diagnostic confirmation
**11**	77	NHL	rituximab, bendamustine 07-11/2019	5	activated	35	65		type 4	5.81	411	1.77	< 500	
**12**	69	multiple myelo		6		0	100			20.1	1030	2.8	positive	

		ma												
**13**	69	T-NHL	CHOP-21 (6x),DHAP (2x)	3		0	100		type 4	6.2	370	1.5	negative	
**14**	70	NHL	rituximab, bendamustine	1		0	100			15.7	800	1,9	20.000.000	
**15**	73	CLL	Multiple chemotherapy, Pembrolizumab, Rituximab	0	lymphocytic	78	10	8	type 1	6.6	520	1.97	positive	
**16**	75	CLL	chemotherapy	0		28	16	9	type 1	5.6	360	1.62	11.650	
**17**	62	CLL	vhemotherapie (R-Benda)	3	lymphocytic	75	6	8	type 2	6	500	n.a.	> 10 Mio	
**18**	39	DLBCL	plasmapheresis	1	lymphocytic	71	15	13	type 1	7.1	610	2.11	1150	
**19**	24	AML	immunoglobulins	14	lymphocytic	97	1	2	type 4	8	760	1.57	15	

Transplant patients

Pat.	Age at diagnosis PML	underlying disease	immunosuppressive therapy	cell count	cytology	lymphocytes (%)	monocytes (%)	others (%)	OCB	Qalbumin	CSF protein (mg/l)	CSF lactate (mmol/l)	CSF JCV PCR (c/ml)	additional findings
1	65	kidney transplant	tacrolimus	8	activated	75	25		type 3	7.47	410	1.35	500	
2	72	liver trasplant	MMF, tacrolimus	1	inconspicuous	n.a.	n.a.		type 1	10.6	750	1.73	negative	histological diagnostic confirmation
3	50	twofold liver transplat, kideny transplant	ciclosporin, MMF	1	inconspicuous	60	40		type 4	3.6	320	1.37	400	
4	54	lung transplant; Hodgkin lymphoma	rituximab, tacrolimus, MMF, ciclosporin	9	activated	76	24		type 2	8.72	578	1.75	19.000	

Others

Pat.	Age at diagnosis PML	underlying disease	immunosuppressive therapy	cell count	cytology	lymphocytes (%)	monocytes (%)	others (%)	OCB	Qalbumin	CSF protein (mg/l)	CSF lactate (mmol/l)	CSF JCV PCR (c/ml)	additional findings
1	77	bronchial carcinoma	unknown chemotherapy	3	lymphocytic	70	30		type 4	6.27	311	n.a.	100.000	
2	68	unknown	none	22	activated	80	14	6	type 2	7.8	574	1.93	1000	
3	50	sarcoidosis	none	4	lytic cells	n.a.	n.a.		type 2	n.a.	440	1.61	n.a.	histological diagnostic confirmation
4	81	unknown	none	1	inconspicuous	60	40		type 4	4.68	340	1.57	11.000	

**5**	36	CVID	none	n.a.	n.a.	n.a.	n.a.	n.a.	n.a.	n.a.	n.a.	n.a.	histological diagnostic confirmation
**6**	52	microscopic polyangiitis	cyclophosphamide (6 cycles), rituximab 05/2014-09/2017	2	inconspicuous	48	52	type 4	10.24	720	1.71	negative	
