# Supplementary material for: Cerebrospinal fluid analysis in 108 patients with progressive multifocal leukoencephalopathy
Source: Fluids Barriers CNS. 2020 Oct 27;17:65. doi: 10.1186/s12987-020-00227-y (PMC7590675; doi:10.1186/s12987-020-00227-y)
Supplement: Supplementary file 2 — Additional file 2. Patients’ characteristics and routine CSF parameter of control group patients. Description: CSF: cerebrospinal fluid; f: female; IIH: idiopathic intracranial hypertension; LP: lumbar puncture; m: male; NPH: normal pressure hydrocephalus; OCB: oligoclonal bands. [file 12987_2020_227_MOESM2_ESM.pdf]

| sex | Age at LP (years) | cell count (cells/µl) | OCB | Qalbumin | CSF protein (mg/l) | CSF lactate (mmol/l) | suspected diagnosis |
|---|---|---|---|---|---|---|---|
| **f** | 25 | 0.3 | type 1 | 2.83 | 244 | 1.57 | IIH |
| **m** | 79 | 0.7 | type 4 | 9.31 | 550 | 2.07 | NPH |
| **m** | 61 | 0.3 | type 1 | 9.95 | 663 | 1.64 | IIH |
| **f** | 56 | 1.0 | type 1 | 3.97 | 318 | 1.58 | NPH |
| **m** | 67 | 2.3 | type 1 | 7.52 | 533 | 1.85 | NPH |
| **f** | 31 | 0.7 | type 4 | 13.63 | 845 | 1.44 | IIH |
| **f** | 48 | 4 | type 3 | 7.6 | 600 | 1.66 | IIH |
| **f** | 41 | 0.7 | type 1 | 6.6 | 507 | 1.44 | IIH |
| **f** | 30 | 0.7 | type 4 | 8.68 | 536 | 1.49 | IIH |
| **f** | 81 | 0.7 | type 4 | 4.26 | 329 | n.a. | NPH |
| **f** | 79 | 1.7 | type 4 | 11.22 | 611 | 1.62 | NPH |
| **m** | 84 | 1.3 | type 4 | 7.31 | 494 | 1.76 | NPH |
| **f** | 35 | 0.3 | type 1 | 5.08 | 364 | 1.40 | IIH |
| **f** | 29 | 2 | type 1 | 4.09 | 312 | 1.40 | IIH |
| **m** | 31 | 0.3 | type 1 | 3.07 | 225 | 1.34 | IIH |
| **f** | 33 | 0.7 | type 1 | 8.53 | 472 | 1.67 | IIH |
| **m** | 31 | 3.3 | type 1 | 2.97 | 273 | 1.55 | NPH |
| **m** | 27 | 0.3 | type 4 | 4.73 | 363 | 1.23 | IIH |
| **f** | 40 | 0.7 | type 1 | 9.09 | 667 | 1.41 | IIH |
| **m** | 65 | 2.3 | type 4 | 6.21 | 447 | 1.36 | IIH |
| **m** | 79 | 0.7 | type 1 | 4.74 | 408 | 1.84 | NPH |
