# Supplementary material for: Cerebrospinal fluid analysis in 108 patients with progressive multifocal leukoencephalopathy
Source: Fluids Barriers CNS. 2020 Oct 27;17:65. doi: 10.1186/s12987-020-00227-y (PMC7590675; doi:10.1186/s12987-020-00227-y)
Supplement: Supplementary file 3 — Additional file 3. Patients’ characteristics and routine CSF parameter of HIV control group patients. Description: CSF: cerebrospinal fluid; f: female; LP: lumbar puncture; m: male; n.a.: not applicable; OCB: oligoclonal bands. The suspected diagnoses as reasons for lumbar punctures included cognitive retardation, suspected encephalitis, suspected vasculitis, unexplained encephalopathy, and seizures. [file 12987_2020_227_MOESM3_ESM.pdf]

sex	Age at LP (years)	cell count (cells/µl)	OCB	Qalbumin	CSF protein (mg/l)	CSF lactate (mmol/l)	additional information
**m**	42	0.3	type 2a	5.46	431	2.45	
**m**	52	5	type 3	6.38	544	2.29	
**m**	43	1.7	type 4	8.29	551	1.53	
**f**	36	1	type 2	3.03	270	n.a.	
**m**	64	1	type 3a	4.44	435	1.65	
**f**	25	0.7	type 3a	5.9	441	1.55	
**m**	50	2	type 2	11.63	734	2.19	
**m**	80	1.3	type 4	5.95	463	1.79	
**f**	43	4	type 3	3.51	307	n.a.	
**m**	26	2	type 3	5.19	394	n.a.	
**f**	54	1	type 3	10.85	614	2.47	
**m**	30	0.3	type 4	8.14	453	1.55	
**m**	30	0.3	type 1	5.94	408	2.25	
**m**	53	1	type 2	2.81	239	n.a.	
**m**	56	16	type 3a	1.4	112	1.54	suspected toxoplasmosis
**m**	83	1	type 3	7.76	552	1.92	
**m**	43	2	type 4	7.56	443	2.58	
**m**	47	1	type 2	6.7	626	1.77	
**m**	69	63	n.a.	10.9	814	1.63	
**w**	42	21	type 3	6.3	1280	2.04	
**w**	51	1	n.a.	4.1	376	1.82	
**w**	62	1	type 3	3.4	245	1.57	
**w**	53	5	type 3	10.3	715	1.74	
**m**	22	4	type 4	7.1	567	1.44	
**m**	47	1	type 2	8.2	661	1.58	
**m**	38	1	type 1	4.4	368	1.36	
**w**	64	5	type 2	7.5	674	1.94	
**w**	44	1	type 2	4.5	424	1.71	
**w**	66	0	type 1	5.1	477	1.69	
**m**	55	0	type 2	8.7	646	2.11	
**w**	25	0	type 1	3.2	269	1.26	
**m**	52	1	type 1	7.6	698	1.63	
**w**	48	3	type 3	9.7	882	1.40	
**m**	69	0	type 3	8.3	668	2.90	
**m**	45	3	type 3	9.7	741	1.67	
**w**	41	1	type 3	1.5	209	1.51	
**m**	50	2	type 1	4.7	352	2.08	
