# Supplementary material for: Cerebrospinal fluid analysis in 108 patients with progressive multifocal leukoencephalopathy
Source: Fluids Barriers CNS. 2020 Oct 27;17:65. doi: 10.1186/s12987-020-00227-y (PMC7590675; doi:10.1186/s12987-020-00227-y)
Supplement: Supplementary file 4 — Additional file 4. Patients’ characteristics and routine CSF parameter of MS control group patients. Description: CSF: cerebrospinal fluid; f: female; LP: lumbar puncture; m: male; n.a.: not applicable; OCB: oligoclonal bands. [file 12987_2020_227_MOESM4_ESM.pdf]

sex	Age at LP (years)	cell count (cells/µl)	OCB	Qalbumin	CSF protein (mg/l)	CSF lactate (mmol/l)
f	43	14	type 2	5.61	428	1.54
m	42	0	type 2	2.7	253	1.6
f	32	3	type 2	3.1	227	2.9
f	54	11	type 2	7.3	489	1.7
m	36	7	type 2	4.35	304	1.94
f	32	2	type 2	4.2	330	1.9
m	40	11	type 2	7.4	551	1,6
f	33	8	type 2	6.9	512	2
m	46	7	type 3	7.4	559	1.6
f	38	6	type 2	5.3	434	3.1
m	30	11	type 2	6.1	512	1.8
f	45	5	type 2	2.3	236	2.65
m	47	10	type 3	16.4	1100	2.4
f	34	6	type 2	2.2	239	1.7
m	39	5	type 2	6.3	449	1.6
f	43	5	type 3	5.4	460	1.7
m	42	0	type 2	2.7	253	1,6
f	35	15	type 2	3.6	253	2.1
m	38	12	type 2a	5.28	368	2.21
f	52	19	type 2	6.1	533	2.1
m	27	30	type 2	5.9	470	1.6
f	42	43	type 3	7.5	610	1.7
m	19	8	type 2	3.9	351	2
f	34	7	type 2	2.9	318	1.7
m	47	1	type 2	7.28	512	1.73
f	60	4	type 3	4.8	343	2.5
m	39	27	type 2	6.2	654	1.8
f	51	1	type 2	5.65	416	1.5
m	35	5	type 2	7	494	1.8
f	48	3	type 2	5.9	572	1.9
f	34	4	type 2	4.7	449	1.6
m	43	5	type 2	7.15	515	1.94
f	59	2	type 2	6.13	440	1.53
m	53	5	type 2	4.3	309	1.3
f	52	0	type 2	9.3	680	1.7
f	40	17	type 2	3.47	321	1.81
f	48	1	type 2	5.4	507	1.9
f	33	10	type 2	3.6	291	1.9
m	33	7	type 2	3.43	275	1.51
f	33	6	type 3	3.8	338	1.8
m	50	7	type 2	4.7	436	1.8
f	44	22	type 2	4.19	371	2.18
m	50	33	type 3	4.33	383	1.73
f	37	5	type 2	3.6	343	2.6
f	33	10	type 2	5.83	437	1.42
f	44	6	type 2	5.7	422	1.6
f	33	3	type 2	4.4	412	1.4

**f**	25	29	type 2	3.4	325	2
**m**	52	5	type 3	8.26	479	1.7
**f**	50	1	type 2	2.6	324	1.3
**f**	44	2	type 2	3.72	318	1.6
**m**	52	4	type 3	11.9	614	2.4
**f**	41	8	type 2	9.2	616	1.7
**f**	51	14	type 2	8.1	576	1.4
**f**	43	14	type 2	5.61	428	1.54
